# Supplementary material for: Splice-Junction-Based Mapping of Alternative Isoforms in the Human Proteome
Source: Cell Rep. Author manuscript; Available in PMC 2020 Jan 15. (PMC6961840; doi:10.1016/j.celrep.2019.11.026)
Supplement: 2 [file NIHMS1546469-supplement-2.zip › DF1/PXD010154/Heart/Heart_78_TPM3_AISEELDHALNDMTSGSQMLKLDK.pdf]

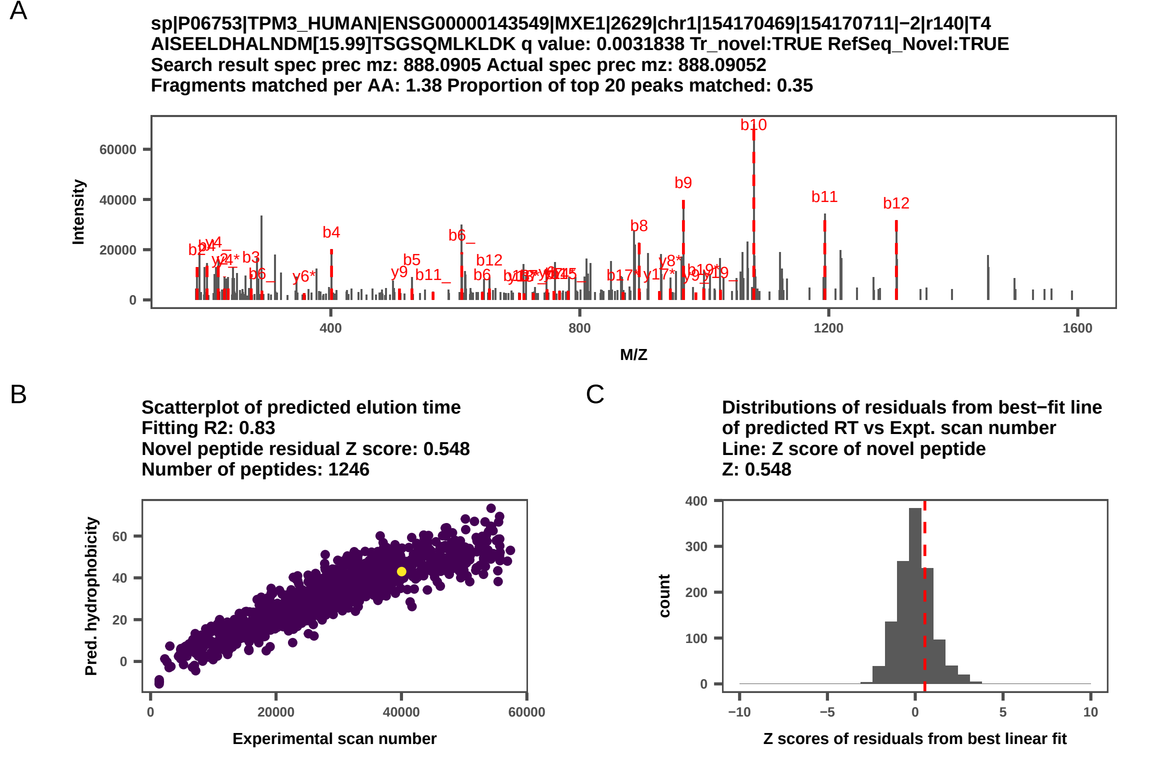
A
sp|P06753|TPM3_HUMAN|ENSG00000143549|MXE1|2629|chr1|154170469|154170711|−2|r140|T4
AISEELDHALNDM[15.99]TSGSQMLKLDK q value: 0.0031838 Tr_novel:TRUE RefSeq_Novel:TRUE
Search result spec prec mz: 888.0905 Actual spec prec mz: 888.09052
Fragments matched per AA: 1.38 Proportion of top 20 peaks matched: 0.35
Intensity
M/Z
b10
b9
b11
b12
b8
b4
B
Scatterplot of predicted elution time
Fitting R2: 0.83
Novel peptide residual Z score: 0.548
Number of peptides: 1246
Pred. hydrophobicity
Experimental scan number
C
Distributions of residuals from best−fit line
of predicted RT vs Expt. scan number
Line: Z score of novel peptide
Z: 0.548
count
Z scores of residuals from best linear fit
